# Genetic and molecular biology of systemic lupus erythematosus among Iranian patients: an overview

**DOI:** 10.1186/s13317-020-00144-y

**Published:** 2021-01-30

**Authors:** Meisam Gachpazan, Iman Akhlaghipour, Hamid Reza Rahimi, Ehsan Saburi, Majid Mojarrad, Mohammad Reza Abbaszadegan, Meysam Moghbeli

**Affiliations:** 1 Department of Medical Genetics and Molecular Medicine, School of Medicine, Mashhad University of Medical Sciences, Mashhad, Iran; 2 Student Research Committee, Faculty of Medicine, Mashhad University of Medical Sciences, Mashhad, Iran

**Keywords:** Systemic lupus erythematosus, Genetic, Diagnosis, Marker, Iran

## Abstract

**Background:**

Systemic lupus erythematosus (SLE) is a clinicopathologically heterogeneous chronic autoimmune disorder affecting different organs and tissues. It has been reported that there is an increasing rate of SLE incidence among Iranian population. Moreover, the Iranian SLE patients have more severe clinical manifestations compared with other countries. Therefore, it is required to introduce novel methods for the early detection of SLE in this population. Various environmental and genetic factors are involved in SLE progression.

**Main body:**

In present review we have summarized all of the reported genes which have been associated with clinicopathological features of SLE among Iranian patients.

**Conclusions:**

Apart from the reported cytokines and chemokines, it was interestingly observed that the apoptosis related genes and non-coding RNAs were the most reported genetic abnormalities associated with SLE progression among Iranians. This review clarifies the genetics and molecular biology of SLE progression among Iranian cases. Moreover, this review paves the way of introducing an efficient panel of genetic markers for the early detection and better management of SLE in this population.

## Background

Autoimmune disorders are associated with immune system attack to the body's own organs, tissues, and cells [1, 2]. They have an increasing frequency in industrialized countries. Systemic lupus erythematosus (SLE) is a heterogeneous autoimmune disorder characterized by antinuclear, anti-double-stranded DNA, and antiphospholipid antibodies [3]. The incidence rate of SLE varies in different geographical regions from 1 to 10 per 100,000 person annually [4]. North America has the highest rate of SLE incidence (23.2/100,000) and prevalence (241/100,000) [5, 6], while Africa and Ukraine have the lowest incidences (0.3/100,000 persons/year) [7, 8]. In Asia, the Chinese and Asian Indians have higher SLE prevalence in comparison with Arabs [9–12]. Various clinical symptoms are observed in SLE patients such as renal failure, arthritis, thrombosis, and neurologic complications. There are different environmental and genetic risk factors associated with SLE etiology. SLE is more prevalent in women (about nine times more than men) and more diagnosed between 15 and 44 years old [4]. Smoking [13], alcohol consumption [14], metals [15], air pollution [16], obesity [17], diet [18], infections [19], pesticides [20], and silica [21] are environmental risk factors associated with SLE. Genetic factors have also important roles during SLE progression which are mainly associated with combined effect of various genes. Single-nucleotide polymorphisms (SNPs) that are associated with SLE pathogenesis are also mainly located in noncoding DNA sequences of immune system genes [22]. The prevalence of SLE in Iran as a middle-east country is reported 40 per 100,000 persons. SLE has more severe symptoms among Iranian patients compared with European Caucasians. It seems that the higher severity of SLE among Iranians can be associated with some environmental risk factors such as ethnic and diet in which Iranians as a non-white population has higher SLE severity compared with European population (white). Moreover, low carbohydrate/fiber, high protein/fat intakes, and micronutrients deficiencies among Iranians can also be associated with high severity of SLE in this population [23–27]. Poor sleep quality and vitamin D deficiency has been reported among Iranian SLE patients [28, 29]. Regarding the severe clinical complications among Iranian SLE patients, it is required to introduce a diagnostic panel of genetic markers for the early detection of SLE. Therefore, in present review we have summarized all of the reported genes with significant effects on SLE progression among Iranian cases. We also categorized them based on their cell and molecular functions to clarify the biology of SLE among Iranian population. Moreover, we categorized the reported factors based on their outcomes into genetic/epigenetic aberrations and cytokines/chemokine abnormalities (Tables 1, 2).

**Table 1:** All of the genetic aberrations with significant effects on clinicopathological features of SLE among Iranian patients

Study (et al.)	Year	Gene	Population	Results
Tahmasebi [36]	2013	IL-1RN	213 NC^a^ 207 SLE^b^	Polymorphism was correlated with SLE progression
Mahmoudi [42]	2014	IL-4	140 NC 59 SLE	Polymorphism was correlated with SLE progression
Mohammadi [47]	2019	IL-10	131 NC 116 SLE	Polymorphism was correlated with SLE progression
Mirkazemi [58]	2013	STAT4	281 NC 280 SLE	Polymorphism was correlated with SLE progression
Alesaeidi [76]	2015	MECP2	392 NC 492 SLE	Polymorphism was correlated with SLE progression
Sahebari [79]	2010	FAS	50 NC 114 SLE	Different serum levels of Fas between cases and controls
Moudi [82]	2013	FAS, FASL	149 NC 106 SLE	Polymorphism was correlated with SLE progression
Araste [86]	2010	FAS	249 NC 212 SLE	Polymorphism was correlated with SLE progression
Fathi [90]	2020	PDCD1	564 NC 253 SLE	Polymorphism was correlated with SLE progression
Mahmoudi [91]	2015	PDCD1	50 NC 202 SLE	Polymorphism was correlated with SLE progression
Rajabi [102]	2012	TNFSF4, TRAF2	57 NC 57 SLE	Increased and decreased levels of TNFSF4 and TRAF2 expressions respectively
Namazi [107]	2017	APRIL	64 NC 60 SLE	Increased serum APRIL levels
Salimi [110]	2018	ERa	186 NC 170 SLE	Polymorphism was correlated with SLE progression
Shojaa [114]	2017	CTLA‐4	304 NC 180 SLE	Polymorphism was correlated with SLE progression
Salimi [118]	2014	XRCC1	180 NC 163 SLE	Polymorphism was correlated with SLE progression
Jahantigh [122]	2015	XRCC5, XRCC7	180 NC 163 SLE	Polymorphism was correlated with SLE progression
Salimi [131]	2016	Osteopontin	180 NC 163 SLE	Polymorphism was correlated with SLE progression
Mirfeizi [135]	2012	UMCP-1	67 SLE	Increased UMCP-1 levels
Noroozinia [143]	2016	CD34	73 SLE	CD34 expression was associated with activity index
Sharifipour [147]	2013	LCN2	52 SLE	Increased urinary LCN2/creatinine level
Bahrehmand [156]	2012	MMP-2	101 NC 109 SLE	Polymorphism was correlated with SLE progression
Bahrami [175]	2020	PTPN22	93 NC 55 SLE	Polymorphism was correlated with SLE progression
Sandoughi [185]	2016	eNOS	194 NC 106 SLE	Polymorphism was correlated with SLE progression
Bahrehmand [197]	2013	PON1	83 NC 109 SLE	Polymorphism was correlated with SLE progression
Tanhapour [199]	2018	ApoE, PON1	117 NC 101 SLE	Polymorphism was correlated with SLE progression
Khoshmirsafa [203]	2019	miR-16, miR-21, and miR-155	30 NC 55 SLE	Increased expression of miR-16, miR-21, and miR-155 while miR‐141 down regulation
Vahed [206]	2018	miR-125a, miR-142-3p, miR-146a	26 NC 26 SLE	Increased circulating miR-125a and miR-146a levels while reduced level of circulating miR-142-3p
Nakhjavani [209]	2019	miR-21, miR-150, miR-423	26 NC 26 SLE	Reduced levels of circulating miR-150 while increased levels of circulating miR-21 and miR-423
Akhtari [214]	2016	KIR, HLA	273 NC 230 SLE	Polymorphism was correlated with SLE progression
Rezaieyazdi [215]	2008	HLA	83 NC 40 SLE	Polymorphism was correlated with SLE progression

^a^Normal control (NC)

^b^Systemic lupus erythematosus (SLE)

**Table 2:** All of the reported cytokines/chemokines abnormalities during SLE progression among Iranian patients

Study (et al.)	Year	Gene	Population	Results
Sedighi [37]	2014	IL-2	73 NC^a^ 73 SLE^b^	IL-2 was significantly correlated with Prednisone consumption
Mohammadi [61]	2019	IL-17	20 NC 40 SLE	Increased IL-17 levels is SLE patients receiving glucocorticoids
Rastin [66]	2016	IL-6‚ IL-17, IFN-γ	40 SLE	IL-17, IL-6, and IFN-γ up regulations
Aghdashi [69]	2013	IL-18	25 NC 25 SLE	Serum levels of IL-18 were correlated with platelet counts and C3 levels
Jafari-Nakhjavani [71]	2016	IL-18	50 NC 113 SLE	Increased serum levels of IL-18
Loghman [73]	2016	Adiponectin	50 SLE	Increased urinary levels of adiponectin
Sahebari [80]	2012	Fas, IL-18	50 NC 114 SLE	Increased serum levels of Fas and IL-18
Hatef [81]	2013	IL-18, Fas	46 NC 32 SLE	Increased serum levels of Fas and IL-18
Abediazar [128]	2019	CXCL10	39 NC 25 SLE	Increased levels of CXCL10
Hajialilo [142]	2018	VCAM-1, ET-1	40 NC 60 SLE	Up regulations of serum VCAM-1 and ET-1
Yazdanpanah [167]	2017	TLR3, TLR7, TLR9	20 NC 20 SLE	TLR7 and TLR9 up-regulations
Mortezagholi [169]	2016	TLR9	38 NC 35 SLE	Increased expression of TLR9

^a^Normal control (NC)

^b^Systemic lupus erythematosus (SLE)

## Main text

### Cytokines

Cytokines are soluble glycoproteins that function in autocrine/paracrine states between leukocytes and other cells which are involved in leukocyte growth and migration [31, 32]. Moreover, various other biological processes such as angiogenesis and inflammation are associated with cytokines production via lymphocytes, monocytes, keratinocytes, and endothelial cells [33]. IL-1 is a pro inflammatory cytokine involved in autoimmune responses [34]. The IL-1 receptor antagonist (IL-1RN) is a suppressor of IL-1 activity [35]. A significant correlation has been observed between IL-1RN rs315952 polymorphism and SLE among Iranian patients in which the CT genotype was protective. Patients with hematological symptoms had significantly higher frequency of rs315952 T allele. There was also a significant decreased frequency of rs315952 CT genotype in SLE cases compared with controls [36]. There was a significant correlation between IL-2 serum level and SLE disease activity among Iranian cases. The serum level of IL-2 was significantly correlated with Prednisone consumption [37].

IL-4 is a pleiotropic cytokine produced by various cells such as T cells, basophils, and NK cells [38, 39] which has pivotal role in regulating the T helper 2 (Th2) development [40, 41]. It has been shown that there were significant increased frequencies of C allele at -33 and -590 as well as T allele at -1098 SNPs among a sample of SLE cases compared with controls. There were also increased frequencies of 33 CC, 590 CC, and 1098 TT genotypes, while decreased frequencies of 33 TC, 590 TC, and 1098 TG genotypes. IL-4 gene polymorphisms may lead to the reduced frequencies of TTC, GCC and TTT haplotypes, while there was significant increased rate of TCC haplotype in SLE cases [42].

IL-10 is a cytokine mainly secreted by monocytes and B lymphocytes which suppresses the other pro-inflammatory cytokines in activated macrophages and T lymphocytes [43, 44]. IL-10 is involved in SLE pathogenesis through induction of B lymphocytes proliferation and autoantibodies production by damaged organs [45, 46]. It has been reported that the GG genotype of IL-10 (1082) and CC genotype of IL-10 (819) polymorphisms were correlated with increased SLE susceptibility among Iranian patients. There were IL-10 plasma up regulations in CC and AA genotype carriers of -592 and -1082, respectively. The CC and TT genotype carriers at − 592 and − 819 regions respectively had also increased SLEDAI score [47]. Disturbed immune tolerance and T/B lymphocytes activation results in production of autoantibodies. V-Set Domain Containing T Cell Activation Inhibitor 1 (VTCN1) is an inhibitor of T cell responses, cell-cycle progression, and cytokine production [48–51] that can be up regulated by IL-10 and IL-6 [52]. The STAT4 is a transcription factor induced by IL-12 and IL-23 which has a pivotal function in Th1 and Th17 differentiation [53–57]. Therefore, STAT4 can also be involved in SLE pathogenesis [57]. It has been reported that there was a significant correlation between rs7574865TT and GT genotypes and risk of SLE in a sample of Iranian subjects [58].

T helper and regulatory T cells are the main regulators of inflammation during SLE progression. Th1 cells related cytokines are associated with cell-mediated immunity [59], whereas Th17 cells are involved in organ damage through IL-17 production [60]. It has been reported that there was increased IL-17 levels in a sample of Iranian SLE patients receiving glucocorticoid treatments compared with newly diagnosed and healthy cases. There was also a significant direct association between IL-17 and IFN-γ plasma levels while a negative association between IL-17 and IL-10 cytokines [61]. Glomerulonephritis is an important organ involvement in SLE which is associated with poor prognosis and end-stage disease [62]. Regulatory T cells have critical role in regulation of unwanted immune responses and can be involved in lupus nephritis (LN) progression [63, 64]. Th17 cells as effector T helper cells have been observed in damaged organs of SLE cases [65]. Up regulations of Th1 and Th17 cytokines induced nephrogenic conditions in LN. It has been reported that there were IL-17, IL-6, and interferon gamma (IFN-γ) up regulations in class IV glomerulonephritis SLE in comparison with non-nephritis SLE subjects in a sample of Iranian population [66].

IL-18 has a pivotal role in progression of cutaneous lupus erythematosus (CLE) and SLE [67, 68]. It has been reported that there were significant direct associations between serum levels of IL-18 and platelet counts among a sample of Iranian SLE patients with high disease activity, while inverse correlation between IL-18 and C3 levels [69]. One of the feasible mechanisms of SLE progression is Th1 and Th2 imbalanced that leads in B lymphocyte cell activity. IL-18 has a key function in Th1 response toward toxic shocks. It induces INF-γ production by T and NK cells and proliferation of activated T cells [70]. It has been shown that there were significant increased serum levels of IL-18 among Iranian SLE cases compared with healthy subjects. Serum levels of IL-18 were also associated with SLE disease activity index (SLEDAI) and high activity indexes. Active SLE patients had also higher levels of IL-18 compared with chronic cases. Moreover, the SLE cases with renal involvement had significantly higher serum level of IL-18 compared with cases without renal complication [71].

Adiponectin is an adipocyte-derived cytokine involved in renal complications of SLE [72]. There were significant increased urinary levels of adiponectin in Iranian SLE patients with renal complication compared with cases lacking renal involvement [73]. IFN-γ is a soluble cytokine produced by various cells such as Th cells, macrophages, and NK cells which is involved in NK induction and leukocyte migration. Methyl CpG binding protein 2 (MECP2) recruits the histone deacetylase to promoter regions of target genes which induces heterochromatin formation and transcriptional inhibition [74]. It can also suppresses the gene expression via DNA methyltransferase1 (DNMT1) recruitment. MECP2 down regulates the IFN-γ secretion by Th cells that results in a partial immune suppression [75]. It has been reported that there were significant correlations between rs1734787 and rs1734791 polymorphisms of MECP2 and SLE progression among Iranian patients in which the C allele of rs1734787 and T allele of rs1734791 polymorphisms increased the SLE risk. Moreover, there were significant frequencies of CTAT and AAAT haplotypes in cases and controls, respectively [76].

### Apoptosis and DNA repair

Fas/APO-1 belongs to the tumor necrosis factor (TNF) family of proteins that play a significant role in cell death, peripheral tolerance, and autoimmune response [77]. FAS is expressed normally at a low level on resting cells, while is highly expressed by activated T cells [78]. A significant different serum levels of Fas has been observed between a sample of Iranian SLE cases and control group [79]. Another study has been reported that there were increased serum levels of Fas and IL-18 in a sample of Iranian SLE patients compared with controls which were also associated with disease activity and erythrocyte sedimentation rate (ESR) [80]. Increased serum Fas and IL-18 levels were also significantly observed in patients with proteinuria in comparison with cases without proteinuria [81]. A significant different frequency of FAS A-670G AA genotype compared with GG genotype has been shown between Iranian SLE patients and controls. The SLE patients had also significantly increased frequency of A allele compared with G allele. Regarding the FASL C-844T polymorphism, CC genotype and C allele were significantly more frequent in SLE patients compared with healthy subjects. The AA/CC genotypes of FAS A-670G/FASL C-844T increased SLE susceptibility more than other genotypes [82]. The interaction of Fas and FasL results in apoptosis [83]. Soluble fas (sFas) is a variant without transmembrane domain [84] which is observed in supernatants of B and T cell lines [85]. The promoter region polymorphisms of the Fas have been assessed among Iranian SLE patients which showed significant higher frequencies of 1377 G allele and GG genotype in patients compared with controls. There were also reduced frequencies of − 1377 − 670 (A-G)/ − 1377 − 670 (A-G) haplotype among patients compared with healthy cases. Moreover, patients had significantly increased levels of sFas and Fas ligand compared with controls. There was lower levels of anti-SSB/La in-670GG genotype carriers. Therefore, Fas promoter polymorphisms were suggested as risk factors of SLE among Iranian patients [86].

Programmed cell death 1 (PD‐1) is an immunosuppressive factor associated with autoimmune disorders [87, 88]. It has a significant role in regulation of T cells function [89]. The correlation between PDCD1 SNPs and SLE progression was assessed among Iranian population. It has been reported that there was significant increased frequency of PD1.5 C/C genotype in SLE patients compared with healthy cases, while the PD1.5 C/T and T/T genotypes frequencies were reduced in SLE patients. There was also significant correlations between GACT and GGCC haplotypes of PDCD1 and SLE susceptibility, while GGCT was protective during SLE progression [90]. Another group has been reported that there was a significant inverse correlation between PD1.1 GG genotype and juvenile-onset SLE (JSLE) susceptibility among a sub population of Iranian cases. The PD-1.1 A allele was also more frequent among cases in comparison with controls [91].

TNF superfamily member 4 (TNFSF4) has critical roles in regulation of T-cell proliferation and activation which promotes CD4 + T cells survival in inflammation sites [92]. It also induces naive CD4 + T cells for the secretion of IL-4, IL-5, and IL-13 [93, 94]. Moreover, TNFSF4 stimulates B-cell proliferation that results in cell hyperactivity in autoimmune disorders [95–97]. The TNF and TNF receptor have important roles in lymphocyte apoptosis during immune regulation [98]. TNF-R signaling is mediated by TNF-R-associated factor 2 (TRAF2) that is an adaptor protein and ubiquitin ligase [99]. TRAF2 is also associated with non-canonical NF-kB pathway through TNF-α [100]. There is an interaction between TNFSF4 and TRAF2 to modulate apoptosis through NF-KB pathway which is involved in T-cell-mediated autoimmunity [101]. There was increased and decreased levels of TNFSF4 and TRAF2 expressions respectively in PBMCs of Iranian SLE patients compared with controls. A positive association was also between TNFSF4 expression levels and atherosclerotic symptoms in SLE patients. TRAF2 down regulation was also associated with renal involvement and atherosclerosis. The SLE cases with severe clinical symptoms had lower levels of TRAF2 expression which showed a negative association between SLEDAI and TRAF2 down regulation [102].

A proliferation-inducing ligand (APRIL) is belonged to the TNF superfamily involved in B lymphocyte proliferation and antibody production [103]. Heparin sulfate have been also reported as APRIL receptor [104–106]. There was significant increased serum APRIL levels in a sample of Iranian children with SLE compared with healthy cases [107]. Estrogen inhibits the apoptosis in PBMCs of SLE patients and ERα up regulation have been observed among SLE cases [108, 109]. It has been reported that the CC/GG and TC/AA genotypes and TT haplotype of ERaPvuII and XbaI polymorphisms were correlated with increased risk of SLE among Iranian subjects [110].

Cytotoxic lymphocyte antigen-4 (CTLA-4) has critical roles in regulation of T cell activation, apoptosis, and peripheral tolerance [111, 112]. CTLA-4 up regulation in active SLE patients shows a key role during SLE progression [113]. There was an association between CTLA4-318C/T polymorphism and SLE pathogenesis among a sub population of Iranian cases in which the CC genotype was significantly correlated with SLE susceptibility, while the CT genotype and T allele were more frequent among healthy cases [114].

Deregulation of DNA repair system results in DNA breaks that produces immunogenic antigens and induces autoimmune response [115]. XRCC1 is one of the members of base excision repair (BER) system [116] involved in repair of DNA damages caused by various factors such as active oxygen and alkylating agents [117]. It has been reported that there were significant decreased frequency of XRCC1 Arg/Gln genotype in a sample of Iranian SLE patients compared with controls which had also decreased frequency in malar rash positive compared with SLE cases without malar marsh [118]. Homologous recombination (HR) and non-homologous end joining (NHEJ) are the main mechanisms of double-strand break (DSB) repairs [119, 120]. Autoantibodies against Ku as one of the members of NHEJ are reported in SLE patients [121]. It has been shown that there were significant correlations between XRCC7 6721G > T and XRCC5 VNTR polymorphisms and SLE susceptibility in a sample of Iranian subjects. The 0R allele of XRCC5 VNTR polymorphism was more frequent in SLE patients in comparison with controls which introduced 0R allele as a risk factor of SLE [122].

### Chemokines and adhesion factors

Lupus nephritis (LN) is observed in about 35% of early diagnosed SLE patients and up to 60% of patients after 10 years [123]. CXCL10 is a chemokine produced by several cells such as fibroblasts and monocytes which is associated with angiogenesis reduction and T cells migration to the inflammatory sites [124, 125]. The CXCL10 up regulation has been observed in autoimmune disorders [126]. The vasculoprotective role of vitamin D is associated with decreased CXCL10 secretion by macrophages in SLE patients [127]. There were significant elevated and decreased levels of CXCL10 and vitamin D respectively in a sample of Iranian SLE patients in comparison with controls and SLE cases without nephritis. CXCL10 was also associated with SLE disease activity index (SLEDAI) and renal activity [128].

Osteopontin (OPN) is a chemokine with pivotal roles in regulation of bone biology, inflammation, and immune response. It induces and suppresses the Th1 and Th2 responses, respectively [129]. CD44 is the most important receptor of OPN to regulate cellular chemotaxis and adhesion [130]. There was a significant increased frequency of OPN rs1126616CT genotype among a group of Iranian LN patients compared with controls. LN cases had also higher frequency of rs1126616TT genotype compared with controls. Moreover, increased serum OPN level was observed in SLE patients with LN and joint complications in comparison with SLE cases without these symptoms [131].

Urinary monocyte chemoattractant protein 1 (UMCP-1) is an efficient marker of renal complication among lupus cases which is expressed by several renal cells such as endothelial and mesangial cells [132, 133]. It is involved in monocyte and T cells recruitment and activation in acute and chronic inflammation [134]. It has been reported that there were significant elevated UMCP-1 levels in a group of Iranian LN patients compared with LN negative cases [135].

VCAM-1 is belonged to the immunoglobulin-like superfamily produced by various cells such as endothelial cells and macrophages, which stimulates leukocytes adhesion to the vascular endothelium [136–139]. Endothelin-1 (ET-1) is also an endothelial cell-derived factor associated with endothelial dysfunction which has a key role during SLE progression [140, 141]. There were significant up regulations of serum VCAM-1 and ET-1 in a sample of Iranian SLE cases compared with healthy subjects [142]. CD34 is an intercellular adhesion factors expressed in various cells such as hematopoietic cells, endothelial cells, and fibroblasts. CD34 expression was observed in all of a sample of Iranian LN patients which had an inverse association with activity index. Therefore, CD34 can be protective in LN cases. High CD34 expression was also observed in patients with higher SBP and lower WBC count [143].

Renal involvement is an important reason of mortality in SLE patients that is still a big challenge of management because of heterogeneity and complicated course [144]. The Lipocalin-2 (LCN2) is a transporter expressed in neutrophils and renal cells that is up regulated during inflammation [145]. LCN2 promotes cell migration through chemokines up regulations in brain in which the LCN2 amplifies neuro inflammation and inflammatory cells recruitment through CXCL10 up regulation in CNS cells [146]. It has been observed that there were increased urinary LCN2/creatinine level in Iranian LN patients compared with cases without nephritis which was also significantly associated with proteinuria [147].

Coronary heart disease (CVD) and stroke are the main reasons of SLE related deaths [148–150]. Matrix metallopeptidases (MMPs) are zinc-dependent enzymes associated with degradation of extracellular matrixes [151, 152]. MMP-2 is produced by macrophages and has critical roles in SLE progression [152–154]. CCL11 and CXCL12 can up regulate the MMP-2 through PI3K/Akt signaling pathway [155]. A significant correlation has been observed between MMP-2 G1575A polymorphism and CVD progression in Iranian SLE patients. Both MMP-2 1575A allele and G/AþA/A genotype increased SLE susceptibility and CVD progression compared with G/G genotype. SLE patients had also significantly increased rate of G1575A allele compared with controls. Moreover, increased serum levels of MMP-2 and neoptrin were observed among SLE patients with CVD in comparison with patients without CVD [156].

### Toll-like receptors

It has been reported that the abnormal induction of innate immunity through toll-like receptors (TLRs) has an important role during SLE progression [157–159]. The nucleic acids and immune factors are the most common auto antigens in SLE patients which promote innate immune responses through TLRs [160, 161]. TLR3, TLR8, and TLR7 are involved in RNA molecules detection, whereas the TLR9 identifies un-methylated CpG islands [160]. Endosomal TLRs can also be associated with recognition of self-nucleic acids produced following tissue damage and infections [162, 163]. TLRs commitment by PAMPs/DAMPS can activate self-reactive B and T cells which promotes the SLE progression [164, 165]. Decreased serum levels of Vitamin D have been observed in active phase of SLE [166]. It has been reported that there were TLR7 and TLR9 up-regulations in the PBMCs of Iranian SLE compared with control cases. Vitamin D3 also reduced the TLR3, TLR7, and TLR9 expressions in PBMCs of SLE cases in comparison with healthy subjects [167]. B cells have critical functions in pathogenesis of SLE in which their deregulation results in production of auto-antibodies [168]. TLR binding with specific ligands up regulate the pro inflammatory cytokines in autoimmune disease [160]. There was significant increased expression of TLR9 in CD4 + ,CD8 + T, and CD19 + B lymphocytes of SLE patients compared with control cases among Iranian population [169].

PTPN22 is a tyrosine phosphatase associated with negative regulation of T-cell activation [170]. It has an important role in up-regulation of type 1 IFNs following TLR binding in myeloid cells that is involved in suppression of inflammatory arthritis [171]. The PTPN22 polymorphisms have been reported in autoimmune disorders such as SLE, type 1 diabetes, and rheumatoid arthritis [172–174]. The rs1310182 AA and rs12760457 TT genotypes of PTPN22 were significantly correlated with PSLE among Iranian patients [175]. NO is a free radical produced by NO synthetases (NOS) [176]. NO has a key role in various cellular processes such as T lymphocyte activation, signal transduction [177], and apoptosis [178]. Endothelial NOS regulates TLR4-mediated IL-6 production through a NO-independent signaling [179].NO production by monocytes plays a pivotal function in T cell deregulation and continuous mitochondrial hyperpolarization in SLE patients [180, 181]. Vascular dysfunction in SLE patients is correlated with anti-endothelial cell antibody (AECA) [182, 183]. NO stimulates the cell death in endothelial cells through AECA [184]. It has been reported that there was a significant correlation between intron 4 VNTR polymorphism of eNOS and SLE progression in a sub population of Iranian patients in which SLE cases had higher frequencies of a allele and ba and aa genotypes compared with controls [185].

### Antioxidant agents

Various environmental risk factors such as UV and xenobiotic compounds have pivotal roles during SLE progression [186]. Deregulation of antioxidant system results in elevated reactive oxygen species (ROS) during SLE progression [187–189]. Glutathione S-transferases (GSTs) are involved in detoxification of carcinogenic compounds through glutathione binding [190, 191]. GSTM1 and GSTP1 are associated with detoxification of polycyclic aromatic hydrocarbons, whereas the GSTT1 detoxifies simple hydrocarbons. They can also reduce the ROS levels which is a critical cell process for DNA maintenances toward oxidative damages [192, 193]. There was a significant different frequency of GSTT1 null genotype between SLE cases and healthy subjects among a sub population of Iranian cases. GSTT1 null/GSTM1null/GSTP1 Ile/Val genotypes increased SLE susceptibility in this population [194]. Paraoxonase-1 (PON1) hydrolyzes lipid peroxides to maintain LDL against the oxidation. PON1 as an antioxidant that reduces the LDL oxidation is a critical regulator of atherosclerosis [195, 196]. It has been observed that there was a correlation between PON1 55 M/M genotype and SLE susceptibility in a sample of Iranian SLE cases. PON1 55 M/M genotype significantly increased the risk of SLE in comparison with L/L genotype carriers. There was also higher frequency of 55 M/M genotype in SLE patients with hypertension compared with cases without hypertension. Since, the M/M genotype carriers had high levels of neopterin and LDL-C, they had increased risk of hypertension [197]. Apolipoprotein E (ApoE) has also key roles in T lymphocyte proliferation and immune responses [198]. It has been observed that the ApoE4 and PON-55M alleles increased SLE susceptibility in a sample of Iranian patients. Neopterin and MDA had also higher serum levels in SLE patients with ApoE ε3/ε4 and ε3/ε3 genotypes in comparison with controls [199].

### Non-coding RNAs

MicroRNAs (miRNAs) are a super family of non-coding RNAs (ncRNAs) with pivotal roles in immune responses and SLE pathogenesis. They are involved in lupus progression through deregulation of lymphocyte function, TLRs, and NF-κB signaling pathway [200]. MicroRNA deregulations in T and B cells have been reported during SLE progression toward LN [201, 202]. There were significant increased expressions of miR-16, miR-21, and miR-155 while miR-141 down regulation in a sample of Iranian SLE patients compared with controls. MiR-21 and miR-155 had significantly higher levels of expressions in active LN compared with inactive LN patients. There was also an inverse association between miR-155 and C3/C4 serum levels [203]. CCL5 is an inflammatory chemokine that can be up regulated in SLE patients following the KLF13 suppression by miR-125a [204]. The miR-142-3p regulates the CD4 + T and CD4 + CD25 + Treg cells functions that can be associated with SLE progression through B cell hyper stimulation [205]. It has been reported that there were increased circulating miR-125a and miR-146a levels among a sub population of Iranian LN cases compared with controls. There was also a reduced level of circulating miR-142-3p in LN patients compared with controls. Moreover, miR-142-3p levels were significantly correlated with disease activity index [206]. MiR-21 and miR-150 are involved in immune responses via targeting PDCD4 and c-MYC, respectively [207, 208]. Reduced levels of circulating miR-150 has been shown among Iranian LN patients which showed active EMT and renal fibrosis. There were also significant increased levels of circulating miR-21 and miR-423 in a sample of Iranian LN patients in comparison with controls [209].

### Human leukocyte antigens

Human leukocyte antigen (HLA) system has a critical role in regulation of innate and adaptive immunity through antigen presentation of intracellular and extracellular peptides. Natural killer (NK) cells regulate the activity of T lymphocytes and dendritic cells and lymphocyte-related autoimmune responses [210, 211]. Killer cell immunoglobulin (Ig)-like receptors (KIR) are important factors expressed by NK cells which identify HLA class I ligands [212, 213]. KIR and HLA polymorphisms were assessed in a sample of Iranian SLE cases that showed reduced frequency of HLA-A-Bw4 in SLE patients. The KIR3DL1þ; HLA-B-Bw4Thr80þ and KIR2DS1þ; HLA-C2þ carriers had significantly higher hematological and renal complications. Male carriers of KIR3DP1þ had also significant increased prevalence of renal disorders [214]. The HLADQB1 variation was also assessed in a sample of Iranian SLE patients and controls that showed a significant correlation between HLADQ6 (*0601–*0609) and SLE. There was also decreased frequency of DQ7 (*0301–*0304) in SLE patients compared with controls. Moreover, high frequency of DQ5-DQ6 was observed in SLE patients. The DQ6 was the common HLA DQB1 allele correlated with SLE susceptibility among Iranians [215].

## Conclusions

SLE is a chronic autoimmune disorder with a rising prevalence among Iranian population. However, there was not any report about the genetics of SLE in this population. Regarding the critical role of genetic factors during SLE progression, it is required to clarify the molecular biology and genetics of SLE. Therefore, we summarized all of the genes associated with clinicopathological features of SLE which have been reported among Iranian patients. For the first time, it was interestingly observed that the apoptotic related genes and non-coding RNAs have critical roles during SLE progression among Iranians. This review paves the way of introducing a diagnostic panel of genetic markers for the early detection and better management of SLE among Iranian population.

## Data Availability

The datasets used and/or analyzed during the current study are available from the corresponding author on reasonable request.

## Abbreviations

SLE: Systemic lupus erythematosus
SNPs: Single-nucleotide polymorphisms
VTCN1: V-set domain containing T cell activation inhibitor 1
IFN-γ: Interferon gamma
SLEDAI: SLE disease activity index
MECP2: Methyl CpG binding protein 2
TNF: Tumor necrosis factor
ESR: Erythrocyte sedimentation rate
JSLE: Juvenile-onset SLE
UMCP-1: Urinary monocyte chemoattractant protein 1
MMPs: Matrix metallopeptidases
TLRs: Toll-like receptors
NOS: NO synthetases
AECA: Anti-endothelial cell antibody
sFas: Soluble fas
PD-1: Programmed cell death 1
TRAF2: TNF-R-associated factor 2
APRIL: A proliferation-inducing ligand
CTLA-4: Cytotoxic lymphocyte antigen-4
BER: Base excision repair
DSBs: Double-strand breaks
HR: Homologous recombination
NHEJ: Non-homologous end joining
IL-1RN: IL-1 receptor antagonist
sIL-2R: Soluble IL-2 receptor
Th2: T helper 2
CLE: Cutaneous lupus erythematosus
IFNγ: Interferon gamma
DNMT1: DNA methyltransferase1
HLA: Human leukocyte antigen
KIR: Killer cell immunoglobulin (Ig)-like receptor
ROS: Reactive oxygen species
GSTs: Glutathione S-transferases
PON1: Paraoxonase-1
ApoE: Apolipoprotein E
LN: Lupus nephritis
OPN: Osteopontin
LCN2: Lipocalin-2
CVD: Coronary heart disease
miRNAs: MicroRNAs
ncRNAs: Non-coding RNAs
